# Supplementary material for: The determination of causality of drug induced liver injury in patients with COVID-19 clinical syndrome
Source: PLoS One. 2022 Sep 1;17(9):e0268705. doi: 10.1371/journal.pone.0268705 (PMC9436150; doi:10.1371/journal.pone.0268705)
Supplement: Attachment — Submitted filename: Manuscript Number PONE-D-21-31507.doc [file pone.0268705.s001.doc]

Dear (Editor) PLOS ONE

The suggestions that we can give to the manuscript with the title "The determination of causality of drug induced liver injury in patients with COVID-19 clinical syndrome”, are :

1. The grammatical errors in the text were quite at large in number.

Authors need to send the manuscript for proofreading and correction.

2. In my opinion, some of the information provided in the Introduction section are more suitable to be included in the Discussion instead.

A lengthy introduction compared to the Discussion might give an impression that available studies in the literature on this matter is more than adequate in contributing the information without the need of the current study.

3. It is better if the authors can provide more updates or additional facts that can be obtained from this study compared to what already been published in the literature, and also presents data from others Asian countries, especially Southeast Asia, as comparison data.
4. As highlighted by the authors, study sample was very small, many patients were excluded.
5. I suggest that the authors display figures in a format that is easier to understand, especially table 4.
6. Reference : correct or cite in full reference numbers 7, 18, 19.

Kurnia Fitri Jamil,

Universitas Syiah Kuala, Indonesia
