# Supplementary material for: The determination of causality of drug induced liver injury in patients with COVID-19 clinical syndrome
Source: PLoS One. 2022 Sep 1;17(9):e0268705. doi: 10.1371/journal.pone.0268705 (PMC9436150; doi:10.1371/journal.pone.0268705)
Supplement: Attachment — Submitted filename: Response to reviewers comments_PLOS ONE.docx [file pone.0268705.s002.docx]

The editor-in-Chief	14/02/2022

Plos One

Dear sir,

**The determination of causality of drug induced liver injury in patients with COVID-19 clinical syndrome**

Please find enclosed our response to reviewers’ comments. We believe that our response and changes made to the manuscript did enhance its scientific message and will be immensely useful to the readers of this journal

Kind regards,

Dr Mohammed I Danjuma

On behalf of other co-authors

**Response to reviewer’s comment**

**Comment from the Editor**

“Please ensure that your manuscript meets PLOS ONE's style requirements, including those for file naming. The PLOS ONE style templates can be found at In general, the response is reasonable. However, several points require further clarification:”

**Authors response**

Editors comment noted with thanks. We have carried out an exhaustive review of the manuscript styling consistent with PLOS ONE editorial policy as advised.

**Reviewers comment**

“At first the mission of this study was very good and interesting because until now the diagnosis of DILI has always been a problem and is often placed at the end after other possible diagnoses have been ruled out. But upon further reading of this study, it was frankly very confusing. There were inconsistencies in the method because it was first said to be a retrospective cohort but later said to be cross sectional. Moreover, if this was done retrospectively, it would be very difficult to screen out DILI cases based on the RUCAM scale, which has many question components. Of course, there will be a lot of recall bias if the incident has passed. The cross-sectional method is not suitable for determining DILI cases. Moreover, when the initial case of COVID occurred, by looking at the increase in the transaminases enzyme, it was very difficult to determine from the start whether the case was DILI or not”

**Authors response**

We have noted reviewers’ comment with thanks. We regret the oversight if there was an interchangeable mention of study design between “cross sectional” and “retrospective” in different sections of the manuscript. Our study design was retrospective and despite its obvious limitations (as ably highlighted by the reviewer), the changing pattern and clinical phenotypes of DILI across different clinical environment and patient settings, meant that a significant proportion of both the foundational and subsequent published work on it (with RUCAM as a causality determination tool) has been through a mixture of both prospective and retrospective study designs (1,2, 3, 4). We do recognize prospective design is more robust in DILI determination and less prone to confounding especially recall bias. That notwithstanding, peculiar circumstances especially in the setting of COVID-19 pandemic meant that the priority in the initial cycle of the pandemic for example was largely driven by the need to develop new therapeutics and prevention strategies with little or no focus on strategies to examine the effects of these therapeutics. Most of the data that recent studies on DILI are examining were the retrospectively collected repository data that accrued during the pandemic; and have since provided an excellent repository platform to both detect emerging “drug signals” but to also investigate various well validated pathways and algorithms which have been in clinical use (such as our study). But we do recognize the legion of retrospective study design limitations, and have acknowledged as much in the main manuscript (highlighted in yellow)

**Reviewer’s comment**

“The grammatical errors in the text were quite at large in number”

**Authors response**

Reviewer’s comment noted with thanks. We regret the oversight. We have exhaustively reviewed the manuscript and have made relevant corrections to spelling mistakes and grammatical errors as advised

**Reviewer’s comment**

“In my opinion, some of the information provided in the Introduction section are more suitable to be included in the Discussion instead.

A lengthy introduction compared to the Discussion might give an impression that available studies in the literature on this matter is more than adequate in contributing the information without the need of the current study”.

**Authors response**

Reviewer’s comment noted with thanks. We regret the oversight. We have re-written the introduction section to reflect the current understanding of uncertainties that still exists in the adjudication of DILI in patients with COVID-19 clinical syndrome.

**Reviewer’s comment**

“it is better if the authors can provide more updates or additional facts that can be obtained from this study compared to what already been published in the literature, and also presents data from others Asian countries, especially Southeast Asia, as comparison data”

**Authors response**

We have noted reviewers’ comment. We have already cited several studies and authorities from Asia and different parts of the world to provide a comparative context to this current work (reference 6 and 9-12). Additionally, DILI adjudication with RUCAM scale in patients with COVI-19 is an emerging challenge and will explain the relatively few studies there are for a robust comparison with ours.

**Reviewer’s comment**

As highlighted by the authors, study sample was very small, many patients were excluded.

**Authors response**

We note reviewers’ comments with thanks. We did acknowledge that one of the limitations of our work is its relatively small sample size for which we have already recommended validation by a larger sample sized prospective patient cohort (highlighted in yellow).

**Reviewer’s comment**

I suggest that the authors display figures in a format that is easier to understand, especially table 4.

**Authors response**

Many thanks for the suggestion. We have additionally carried a pictorial representation of the data in table 4 in form of Bar chart

**Reviewer’s comment**

“Reference: correct or cite in full reference numbers 7, 18, 19.”

**Authors response**

We noted reviewers’ comment with thanks and regret the oversight regarding the highlighted references. We have amended those references as appropriate.

**References**

1. Abid A, Subhani F, Kayani F, Awan S, Abid S. Drug induced liver injury is associated with high mortality-A study from a tertiary care hospital in Pakistan. **PLoS One**. 2020 Apr 10;15(4)
2. Chen F, Chen W, Chen J, et al. Clinical features and risk factors of COVID-19-associated liver injury and function: A retrospective analysis of 830 cases. Ann Hepatol. 2021; 21:100267
3. Danan G, Teschke R. Roussel Uclaf Causality Assessment Method for Drug-Induced Liver Injury: Present and Future. Front Pharmacol. 2019; 10:853. Published 2019 Jul 29. doi:10.3389/fphar.2019.00853
4. Cunningham M, Iafolla M, Kanjanapan Y, Cerocchi O, Butler M, Siu LL, et al. (2021) Evaluation of liver enzyme elevations and hepatotoxicity in patients treated with checkpoint inhibitor immunotherapy. **PLoS ONE** 16(6): e0253070
